# Altered LINE-1 Methylation in Mothers of Children with Down Syndrome

**DOI:** 10.1371/journal.pone.0127423

**Published:** 2015-05-27

**Authors:** Ivana Babić Božović, Aleksandra Stanković, Maja Živković, Jadranka Vraneković, Miljenko Kapović, Bojana Brajenović-Milić

**Affiliations:** 1 Department of Biology and Medical Genetics, School of Medicine, University of Rijeka, Rijeka, Croatia; 2 Vinča Institute of Nuclear Sciences, Laboratory for Radiobiology and Molecular Genetics, University of Belgrade, Belgrade, Serbia; Duke University, UNITED STATES

## Abstract

Down syndrome (DS, also known as trisomy 21) most often results from chromosomal nondisjunction during oogenesis. Numerous studies sustain a causal link between global DNA hypomethylation and genetic instability. It has been suggested that DNA hypomethylation might affect the structure and dynamics of chromatin regions that are critical for chromosome stability and segregation, thus favouring chromosomal nondisjunction during meiosis. Maternal global DNA hypomethylation has not yet been analyzed as a potential risk factor for chromosome 21 nondisjunction. This study aimed to asses the risk for DS in association with maternal global DNA methylation and the impact of endogenous and exogenous factors that reportedly influence DNA methylation status. Global DNA methylation was analyzed in peripheral blood lymphocytes by quantifying LINE-1 methylation using the MethyLight method. Levels of global DNA methylation were significantly lower among mothers of children with maternally derived trisomy 21 than among control mothers (P = 0.000). The combination of *MTHFR* C677T genotype and diet significantly influenced global DNA methylation (R^2^ = 4.5%, P = 0.046). The lowest values of global DNA methylation were observed in mothers with *MTHFR* 677 CT+TT genotype and low dietary folate. Although our findings revealed an association between maternal global DNA hypomethylation and trisomy 21 of maternal origin, further progress and final conclusions regarding the role of global DNA methylation and the occurrence of trisomy 21 are facing major challenges.

## Introduction

DNA methylation is one of the most important epigenetic factors in the regulation of gene expression. It is required in many processes, such as embryogenesis, gametogenesis, and the silencing of repetitive DNA elements [1]. The patterns of DNA methylation are non-random, well regulated, and tissue-specific [2]. DNA methylation in white blood cells (WBC) may be influenced by certain factors, which include demographics (age, sex, race), behavioural risk factors [cigarette smoking, alcohol intake, body mass index (BMI), and dietary habits such as folate intake], and polymorphism of the methylenetetrahydrofolate reductase gene (*MTHFR* C677T) [3–10]. Aberrant methylation patterns, including global DNA hypomethylation and gene-specific hypermethylation, have been reported to alter gene expression and have been implicated in various human diseases [11–14]. Gene-specific hypermethylation is linked to gene silencing, while global hypomethylation of DNA has been reported to contribute to chromosome instability and to alter gene expression [15,16]. Global DNA hypomethylation occurs mainly at heavily methylated noncoding regions of DNA, particularly repeat sequences and transposable elements [13,14]. It was reported that methylation of Long Interspersed Nucleotide Element-1 (LINE-1) correlates with global DNA methylation, and that the methylation status of LINE-1 in WBC is a potential biomarker in a variety of diseases [8,17–19]. LINE-1 is the largest member of the LINE family and constitutes 17% of the human genome; it has been estimated that more than 500,000 LINE-1 elements are dispersed throughout the genome [20]. Recently, the MethyLight methodology was developed and validated for quantification of LINE-1 methylation [17].

Down syndrome (DS), or trisomy 21 (MIM 190685), is one of the most common chromosomal abnormalities, with a prevalence of 1 in 1000 live births [21]. Trisomy 21 usually results from chromosomal nondisjunction during oogenesis (90%-95%), particularly during the first meiotic division (70%-85%) [22,23]. It has been suggested that DNA hypomethylation might affect chromatin structure in subtelomeric and pericentromeric regions that are critical for chromosome stability and segregation, thus favouring chromosome 21 nondisjunction during meiosis [24]. In addition, maternal age and altered genetic recombination, which are well established risk factors for DS, have been linked with altered DNA methylation patterns [25–29]. However, global DNA methylation has not yet been quantified in any population of mothers of children with DS.

The aims of the present study were to asses (i) the risk for DS in association with maternal global DNA methylation of LINE-1 in WBC, and (ii) the impact of endogenous and exogenous factors that reportedly influence DNA methylation status. Those factors were *MTHFR* C677T polymorphism, maternal age, BMI, dietary intake of folate, cigarette smoking, alcohol intake, and medication use.

## Materials and Methods

### Study population

A total of 94 mothers of children with DS with full trisomy 21 were enrolled in this study. The karyotypes of the parents were confirmed as normal. Only mothers of children with maternally derived trisomy 21 were included. The control group consisted of mothers of healthy children with no personal or family history of DS or other aneuploidy (n = 100). Blood samples of cases and control mothers were collected in collaboration with DS associations from larger cities in Croatia (Rijeka, Zagreb, Pula, Zadar, Split, Karlovac, Čakovec, and Osijek). The control and case groups were age-matched and of the same ethnicity (white). Maternal age was calculated as the age of the mother at the time of sampling. All study protocols were reviewed by the Ethics Committee of the School of Medicine, University of Rijeka. All participants provided written informed consent prior to participation in the study. Before the sampling, study participants were asked to complete a specially created questionnaire that included demographic data, weight and height (for BMI calculation), intake of folate-rich foods, cigarette smoking, alcohol intake, and medication use. The questionnaire was adapted from a food frequency questionnaire validated for the Croatian women [30].

### Genetic analysis

Genomic DNA was isolated from frozen blood samples using the QIAamp DNA Blood FlexiGene DNA Kit (Qiagen, Hilden, Germany). The parental origin of trisomy 21 was determined as previously described [22]. Genomic DNA was quantified using a spectrophotometer according to the manufacturer's instructions (BioMateTM3, Thermo Electron Corporation, USA). The *MTHFR* C677T polymorphism analysis was performed using polymerase chain reaction–restriction fragment length polymorphism (PCR-RFLP) [31].

Using the EpiTec Bisulfite Kit (Qiagen, Hilden, Germany), 500 ng of genomic DNA underwent sodium bisulphite modification. The converted DNA was resuspended in 30 μl TE buffer. Bisulphite-treated DNA was diluted 10× and 8.18 μl was used for each real-time PCR reaction. All samples were stored at -20°C until they were ready for analysis. All methylation analysis was performed within 1 month after the bisulphite modification of the DNA.

Global DNA methylation was analysed by quantifying LINE-1 methylation using MethyLight methodology [17]. The performance characteristics of the MethyLight assay, including precision and reproducibility have been well described [8,17,32–34]. The PCR primers and probes for LINE-1 (Genebank Accession Number X58 075) are presented in Table 1. The LINE-1 primers lack CpG sites; therefore, two specific TaqMan MGB probes (corresponding to the methylated and unmethylated LINE-1 sequence after bisulphite treatment) were used in real-time PCR (Applied Biosystems, Forest City, CA, USA). The VIC-labelled probe hybridizes to the sequence of the methylated LINE-1 allele, and the FAM-labelled probe hybridizes to the sequence of the unmethylated LINE-1 allele. The probe sequences cover multiple CpG sites within the LINE-1 repetitive sequences, which reflect genome-wide methylation density. PCR reactions were performed with a final reaction volume of 25 μl in sealed 96-well plates on the ABI 7500 Real-Time PCR System (Applied Biosystems, Forest City, CA, USA). PCR reactions contained 12.5 μl TaqMan Universal Master Mix, 2.5 μM of each of the LINE-1 primers, and 150 nM of each TaqMan MGB probe. The amount of resulting fluorescence detected, either VIC or FAM, is directly proportional to the amount of PCR product generated from methylated or unmethylated DNA, respectively [32]. This experimental design allowed the analysis of only fully methylated and unmethylated alleles in the LINE-1 repetitive element. If partial methylation occurs at the level of probe discrimination, the sequence derived after bisulphite conversion is not recognized by either of the two probes. For normalization of DNA input, an Alu-based real-time PCR control reaction was performed in parallel with each LINE-1 real-time PCR reaction, as previously described [17]. Primers and probes for Alu sequences (ALU-C4) (Applied Biosystems, Forest City, CA, USA) are shown in Table 1. ALU-C4 PCR reactions contained 12.5 μl TaqMan Universal Master Mix, 0.3 μM of each of the primers, and 100 nM of TaqMan MGB probe corresponding to the ALU-C4 sequence after bisulphite treatment. The cycle conditions for the LINE-1 and ALU-C4 assay were as previously described [8,17]. Real-time PCR was performed in duplicate for each sample.

**Table 1:** Real-time PCR primers and probes.

**Primer**	Primer sequence
Line-1 F	*5′TTATTAGGGAGTGTTAGATAGTGGG3′*
Line-1 R	*5′CCTCTAAACCAAATATAAAAT ATAATCTC3′;*
ALU-C4 F	*5’GGTTAGGTATAGTGGTTTATATTTGTAATTTTAGTA3’*
ALU-C4 R	*5’ATTAACTAAACTAATCTTAAACTCCTAACCTCA3’*
**Probe**	Probe sequence
Line-1 met	VIC–TACTTCGACTCGCGCACGATA-MBG3'
Line-1 unmet	6FAM–CCTACTTCAACTCACACA-MBG3'
ALU-C4	6FAM-CCTACCTTAACCTCCC-MGB3'

The MethyLight data specific for methylated repetitive elements were expressed as percent of methylated reference (PMR) values, and the levels of unmethylated repetitive elements were expressed as percent of unmethylated reference (PUR) values [17,32]. These values were calculated as previously described, with the following changes. The standard curve was established using EpiTect Methylated Control DNA and EpiTect Unmethylated Control DNA (Qiagen, Hilden, Germany), which were mixed to contain 100%, 90%, 75%, 50%, 25%, 10%, and 0% methylated DNA. The mixtures of methylated and unmethylated DNA were diluted 10x and run in each real-time PCR plate with experimental samples. After PCR amplification, data were read using the SDS 1.4.0 software (Applied Biosystems, Forest City, CA, USA). Global DNA methylation was determined using the formula for absolute quantification, which was modified in the sense that PMR and PUR values were incorporated, because those are corrected in relation to the DNA input. Therefore, the final percentage of global DNA methylation was calculated according to the formula PMR/(PMR+PUR) × 100.

### Analysis of the impact of endogenous and exogenous factors on global DNA methylation

Factors included in the analysis were maternal age and BMI as continuous variables, and cigarette smoking, alcohol intake, and medication use as categorical variables. Maternal age was also studied as a categorical variable; two age groups were defined: 35 years of age or younger (≤35 years) and older than 35 years of age (>35 years). This age limit was set because the risk of having a child with DS after 35 years of age rises proportionately with maternal age [26,35]. Because of the complex relationships between genetic variants in the folate pathway and diet, the analysis also included the variable “*MTHFR* C677T genotype/diet”, which represented a combination of *MTHFR* C677T polymorphism and dietary folate intake (rich or poor) [9,10,36,37]. Folate-rich diet implied that mothers consumed at least three folate-rich foods (green leafy vegetables, legumes, liver of veal, fruit, corn flakes, muesli), at least 2–3 times each week. Any lower intake was defined as a low-folate diet. Mothers who consumed one glass of wine or one glass of beer or one small glass of strong alcohol drink, at least one time per week or more often, were defined as consumers. Those who currently smoked cigarettes daily or occasionally were clasified as smokers. Stepwise regression was first performed in all studied mothers (cases and controls together; n = 184), and then in each group separately.

### Statistical analysis

The χ2 test and Fisher's test were used to assess deviations from Hardy–Weinberg equilibrium and to compare allelic and genotypic frequencies between groups. LINE-1 methylation values for cases and controls were compared using a Mann–Whitney U test. The statistical power of 97% was calculated according to Lehmann [38]. Multivariable logistic regression was used to determine the odds ratio and 95% confidence interval for the association between LINE-1 methylation and case-control status while adjusting for BMI and dietary folate intake. The stepwise regression analysis was used to assess the influence of endogenous and exogenous factors, including *MTHFR* C677T polymorphism, age, BMI, dietary intake of folate, smoking, alcohol intake, and medication use, as predictors of global DNA methylation. Kruskal–Wallis ANOVA was used to establish the association of continuous variables with skewed distribution and global DNA methylation. P<0.05 was the defined threshold for statistical significance. Statistica for Windows 10.0 (StatSoft, Tulsa, OK, USA) was used to analyse the data.

## Results

The study included 94 mothers of children with DS of maternal origin and 100 mothers of healthy children with no personal or family history of DS or other aneuploidy. General characteristics of study participants are presented in Table 2. Complete data from questionnaires was available for 95.7% of control and 94% of case mothers; maternal age was the only available parameter for the remaining mothers (controls: n = 4 and cases: n = 6). BMI was significantly higher among case mothers than among control mothers. The frequency of folate-rich food intake was significantly lower in mothers of children with DS than in control mothers (P<0.001; Table 2).

**Table 2:** General characteristics of mothers of children with DS and control mothers of healthy children.

	Cases	Controls	P
Age, years*	39.5 [18–64]	39 [23–65]	0.942
BMI*	25.01 [17.19–39.68]	23.48 [18.07–34.60]	0.007
Diet: N** (%)			
Folate-rich	37 (41)	66 (70)	0.000
Low-folate	53 (59)	28 (30)	
Smoking: N** (%)			
No	60 (67)	62 (66)	0.957
Yes	30 (33)	32 (34)	
Alcohol intake: N** (%)			
No	69 (77)	78 (83)	0.188
Yes	21 (23)	16 (17)	
Medication use: N** (%)			
No	85 (94)	83 (88)	0.111
Yes	5 (6)	12)	

*Median [min—max]

**Mothers of children with DS (N = 90); control mothers (N = 94)

Genotypes and allelic distribution of *MTHFR* C677T in cases and controls were in agreement with Hardy–Weinberg equilibrium. The frequencies of *MTHFR* C677T genotypes and alleles did not not differ significantly between those two groups (P>0.05; Table 3).

**Table 3:** Allelic and genotypic frequencies of *MTHFR* C677T polymorphism.

*MTHFR* C677T	Cases* N (%)	Controls** N (%)	OR (95%CI)	P
Genotype				
CC	46 (49)	49 (49)	Reference	
CT	38 (40)	42 (42)	1.04 (0.57–1.88)	0.903
TT	10 (11)	9 (9)	0.84 (0.32–2.27)	0.738
CT+TT	48 (51)	51 (51)	1.00 (0.57–1.75)	0.993
Alelle				
C	130 (69)	140 (70)	Reference	
T	58 (31)	60 (30)	0.96 (0.62–1.48)	0.856

*Mothers of children with DS; N = 94

**Control mothers; N = 100

### Global DNA methylation

**Fig 1:**
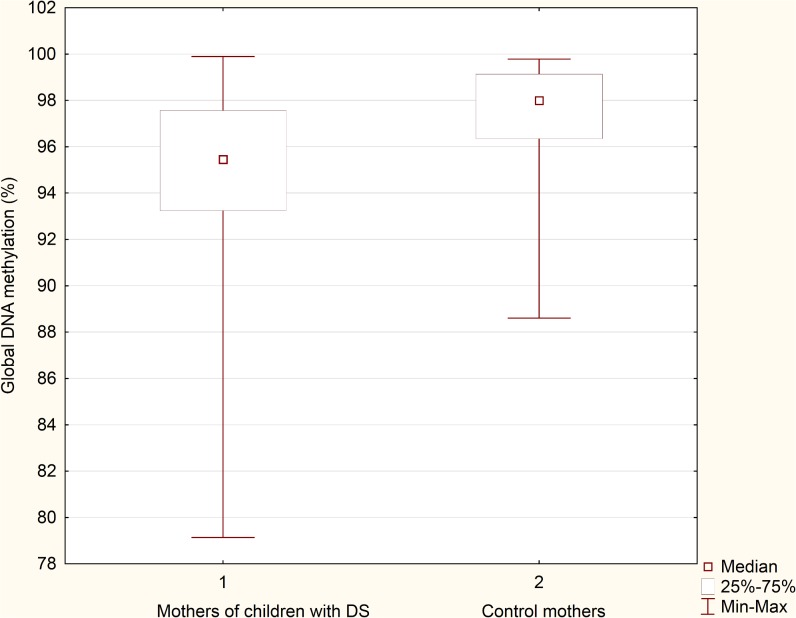
Global DNA methylation in mothers of DS children and control mothers

Global DNA methylation was significantly lower in mothers of children with DS (median: 95.45%; min-max: 79.13%-99.90%) than in control mothers (median: 97.99%; min-max: 88.61%-99.78%) (P = 0.000; Fig 1). After adjusting for BMI and dietary folate intake, LINE-1 methylation was significantly lower among mothers of children with DS (OR = 0.83, 95% CI: 0.733–0.928, P = 0.001).

### The impact of endogenous and exogenous factors on global DNA methylation

Among all studied mothers, group affiliation, *MTHFR* C677T genotype/diet combination and age group were identified as statistically significant predictors of global DNA methylation values (Table 4). Case mothers were significantly more likely to have lower levels of global DNA methylation compared to control mothers (β = 0.256; R^2^ = 0.102; P = 0.000). CC genotype in combination with a folate-rich diet was significantly associated with higher values of global DNA methylation, and the CT+TT genotype and low-folate diet were associated with lower values (β = 0.207; R^2^ = 0.032; P = 0.011). Values of global DNA methylation in case mothers as a function of the *MTHFR* C677T genotype/diet combination are presented in Table 5. The lowest values of global DNA methylation were observed in mothers with CT+TT genotype and a low-folate diet. Mothers >35 years were significantly more likely to have lower levels of global DNA methylation compared to mothers ≤35 years (β = -0.164; R^2^ = 0.026; P = 0.018). Thirty-one percent of mothers (n = 61) were older than 35 years and 69% (n = 133) of mothers were younger than 35 years. Mothers >35 years had significantly lower levels of global DNA methylation (median: 96,60%; min-max: 79,13%- 99,90%) than mothers ≤35 years (median: 97,81%; min-max: 90,56%- 99,78%) (P = 0,016).

**Table 4:** Stepwise regression summary for global DNA methylation in all studied mothers.

All mothers (Cases and controls) (N = 184)				
Dependent variable	Predictor variable	β	Multiple R^2^ change	P
Global DNA methylation	Affiliation to a group cases/controls	0.256	0.102	0.000
	*MTHFR* C677T genotype/diet	0.207	0.032	0.011
	Age group	-0.164	0.026	0.018

Criteria used for independent variable’s entry or removal: F to enter = 4, F to remove = 1.

**Table 5:** Global DNA methylation in all studied mothers depending on the combination *MTHFR* C677T genotype/diet.

All mothers (Cases and controls): N = 184	N (%)	Global DNA methylation (%)*
*MTHFR* C677T genotype/diet		
CC/folate rich diet**	53 (29)	97.85 [89.21–99.75]
CT+TT/folate rich diet	50 (27)	96.96 [89.98–99.90]
CC/folate poor diet	35 (19)	96.53 [88.61–99.78]
CT+TT/folate poor diet**	45 (25)	95.94 [79.13–99.77]

*Median [min—max]

**P = 0,031

Among mothers of DS children, 4.5% of variance in global DNA methylation can be predicted by the *MTHFR* C677T genotype/diet combination (β = 0.588; R^2^ = 0.045; P = 0.046). The lowest values of global DNA methylation were observed in mothers with CT+TT genotype and a low-folate diet (Table 6).

**Table 6:** Global DNA methylation in mothers of children with DS depending on the *MTHFR* C677T genotype/diet.

Mothers of children with DS: N = 90	N (%)	Global DNA methylation (%)*
*MTHFR* C677T genotype/diet		
CC/folate rich diet	21 (23)	96.42 [90.70–99.73]
CT+TT/folate rich diet	16 (18)	94.72 [90.56–99.90]
CC/folate poor diet	22 (25)	95.09 [91.36–98.95]
CT+TT/folate poor diet	31 (34)	94.30 [79.13–99.48]

*Median [min—max]

P = 0.383

Among control mothers, the only statistically significant predictor of global DNA methylation was maternal age group (β = -0.248; R^2^ = 0.062; P = 0.016). Thirty-four percent of mothers (n = 34) were older than 35 years and 66% (n = 66) were younger than 35 years. Mothers >35 years had significantly lower levels of global DNA methylation (median: 97.74%; min-max: 88.61%-99.75%) than mothers ≤35 years (median: 98.82%; min-max: 90.85%-99.78%) (P = 0.003). Mothers of DS children had significantly lower levels of global DNA methylation than control mothers >35 years (P = 0.001) and control mothers ≤35 years (P = 0.000).

Other analysed factors, including cigarette smoking, alcohol intake, medication use and BMI exhibited no association with methylation status, nether in case (P>0.05) nor in control mothers (P>0.05).

## Discussion

To the best of our knowledge, this is the first study to quantify and analyse global DNA methylation in mothers of children with DS. Our results revealed significantly lower level of LINE-1 methylation, a surrogate marker of global DNA methylation, in mothers of children with DS than in mothers of healthy children (95.45% and 97.99%, respectively). The obtained values of global DNA methylation were in accordance with other reports and range from 79% to 100% [8,17,39]. Two strengths of the present study should be emphasised. First, all participants were of the same ethnicity, which is important because an effect of race on DNA methylation has been reported [6]. Second, all trisomy 21 cases were of maternal origin, which allowed us to explore the real-life effect of DNA methylation as a maternal risk factor for DS. Namely, the causal link between DNA hypomethylation and genetic instability has been proposed by numerous previously published studies. DNA hypomethylation has been associated with aneuploidy in human cancer cell lines, with abnormal chromosomal structures in cells from patients with immunodeficiency, centromeric instability and facial anomalies syndrome, and with increased rate of aneuploidy, polyploidy, and chromosomal aberrations in cells treated with the demethylating agents [15,40–45]. Further, the values of global DNA methylation were associated with combination of the *MTHFR* C677T polymorphism and dietary folate intake (*MTHFR* C677T genotype/diet). The *MTHFR* gene encodes the MTHFR enzyme, which catalyses the conversion of 5,10-methylenetertahydrofolate to 5-methylene tetrahydrofolate, the main donor of methyl groups for numerous methylation reactions, including DNA methylation [46]. *MTHFR* C677T polymorphism reduces enzyme activity by approximately 35% in individuals with the CT genotype, and by 70% in individuals with the TT genotype [36]. However, increased folate intake can neutralize the polymorphism’s metabolic impact and restore normal enzyme activity [37,47]. Moreover, studies that quantitatively measured global DNA methylation in WBC of healthy persons found that individuals with the *MTHFR* 677TT genotype and a lower concentration of folate in the blood have significantly lower global DNA methylation than individuals with CT or CC genotypes [9,10]. Our results show that *MTHFR* C677T genotype/diet combination was a significant predictor of global DNA methylation variance in mothers of children with trisomy 21. Although not statistically significant, values of global DNA methylation in mothers of DS children were stratified according to the combination *MTHFR* C677T genotype/diet, wherein the lowest values were observed in mothers with the CT+TT genotype and a low-folate diet, and the highest levels of global DNA methylation were observed in mothers with the CC genotype and a folate-rich diet. Our findings support the initial hypothesis of James et al. that maternal *MTHFR* C677T polymorphism may lead to the hypomethylation of chromosomal regions which are essential for their stability and segregation, and therefore represent a potential maternal risk factor for chromosome 21 nondisjunction [24]. They were the first who analysed *MTHFR* C677T polymorphism as a maternal risk factor for trisomy 21, and found that mothers of children with DS have mildly elevated plasma homocysteine levels and a 2.6-fold increased frequency of *MTHFR* C677T polymorphism in one or both alleles. Numerous studies have been conducted to address this issue, as reviewed by Coppede et al [48]. Discrepancies between different reports imply that the relationship between folate metabolism and chromosome 21 nondisjunction is much more complex, and indicate the importance of other factors that might affect folate metabolism and modify the maternal risk for trisomy 21, including but not limited to maternal age and ethnicity, dietary habits, alcohol consumption, and pregnancy [48]. Based on all of the abovementioned, it is possible to assume that CT+TT genotype and a low-folate diet could be involved in reducing the values of global DNA methylation, and may contribute to the genome instability or nondisjunction of chromosome pair 21 during oogenesis. In fact, the global DNA hypomethylation that we observed in mothers of children with DS may reflect the hypomethylation of centromeric and pericentromeric DNA regions. Studies have shown that DNA methylation plays an important role in modulating the structure and dynamics of heterochromatin [49]. The assembly of heterochromatin is likely to contribute to various chromatin-dependent processes, as well as the functional organization of centromeres and telomeres [49–53]. The hypomethylation of centromeric and pericentromeric DNA regions could lead to the reactivation of epigenetically silenced repetitive elements or the failure of correct kinetochore assembly, microtubule attachment, or sister-chromatid cohesion, which have been associated with irregular chromosomal separation [52,54–60]. Alteration of telomere methylation patterns might also compromise chromosomal stability, through telomere-length deregulation and increased homologous recombination [61]. In addition, DNA methylation may be one factor that affects the recombination pattern between homologues, which (aside from maternal age) is also a well-established risk factor for chromosome 21 nondisjunction [25–28]. It would be interesting to determine whether the global DNA hypomethylation observed in mothers of DS children might have altered recombination pattern, thus favouring the improper segregation of chromosome 21. Although recombination pattern was not established in our case mothers, the relationship between DNA methylation status and recombination pattern certainly represents a promising subject that will be investigated in our future studies.

The age-dependent effect on DNA methylation has been intensively investigated, and it has been suggested that global DNA methylation reduces with increased age [29]. The mechanism underlying DNA hypomethylation is still unclear; however, several factors may trigger and contribute to the loss of genomic methylation, including the activity and expression of DNA methyltransferase, the status of one-carbon metabolism, and the integrity of the genome [62]. Therefore, DNA hypomethylation that occurs during normal ageing appears to be a possible risk factor contributing to the development of age-related human diseases, including cancer, atherosclerosis, Alzheimer’s disease, psychiatric disorders, and autoimmune pathologies [11–14,29]. However, determinants of blood DNA methylation in repetitive elements in healthy individuals are still largely unexplored, and inconsistent patterns have been reported because of the challenges of interpreting results from different assays and from different sources of DNA. All studies investigating ageing and Alu methylation support a lower level of DNA methylation of this repetitive element with increasing age [4,5]. In contrast, only one study found a correlation between lower levels of LINE-1 methylation and increasing age, while most studies did not report an age-dependent effect on blood LINE-1 methylation [4,5]. In our study, LINE-1 methylation did not correlate significantly with maternal age in either of studied groups. However, it is worth noting that control mothers older than 35 years of age exhibited significantly lower levels of global DNA methylation than mothers younger than 35 years of age. This finding implies that advanced maternal age is a contributing factor for global DNA hypomethylation. However, global DNA methylation in mothers of DS children was significantly lower, even compared with the group of control mothers older than 35 years of age. Therefore, it can be assumed that, in mothers of children with trisomy 21 of maternal origin, global DNA hypomethylation is caused by numerous factors, including genetic factors (*MTHFR* 677 CT+TT), low-folate diet, and advanced age.

Regarding the other factors analysed, cigarette smoking, alcohol intake and BMI exhibited no association with LINE-1 methylation status in WBC, which is in accordance with other reports [4–6,63]. Although few studies reported significant associations between LINE-1 methylation and BMI, the direction of change is not consistent [64,65]. The effect of medications on global DNA methylation was also not statistically significant. Only 6% of case mothers and 12% of control mothers were taking medications that belonged to several different therapeutic groups. At the moment we cannot discuss the association between certain medications and global DNA methylation.

In conclusion, our results revealed significantly lower levels of global DNA methylation in mothers of DS children than in mothers of healthy children. Global DNA hypomethylation is significantly associated with *MTHFR* 677 CT+TT genotype and low dietary folate in combination, as well as with ageing. Some limitations in our study should be considered. Global DNA methylation values were not measured during oogenesis, when changes in methylation status would be expected to have the greatest effect. Unfortunately, this information is essentially impossible to obtain, since oogenesis begins during embryonic development of the mother. However, determination of global DNA methylation status may have identified women who were persistently affected by the same exogenous and particularly endogenous factors which lead to global DNA hypomethylation. Based on numerous reports about the association between global DNA hypomethylation and genome instability it could be presumed that maternal global DNA hypomethylation might be involved in chromosome 21 nondisjunction. More extensive work should be done to give the answer to this question.

## References

[1] Li E. Chromatin modification and epigenetic reprogramming in mammalian development. Nat Rev Genet. 2002;3: 662–673. 12209141 10.1038/nrg887

[2] Bestor TH. The DNA methyltransferases of mammals. Hum Mol Genet. 2000;9: 2395–2402. 11005794 10.1093/hmg/9.16.2395

[3] Attig L, Gabory A, Junien C. Nutritional developmental epigenomics: immediate and long-lasting effects. Proc Nutr Soc. 2010;69: 221–231. 10.1017/S002966511000008X 20202279

[4] Terry MB, Delgado-Cruzata L, Vin-Raviv N, Wu HC, Santella RM. DNA methylation in white blood cells: association with risk factors in epidemiologic studies. Epigenetics. 2011;6: 828–837. 21636973 10.4161/epi.6.7.16500 PMC3154425

[5] Zhu ZZ, Hou L, Bollati V, Tarantini L, Marinelli B, Cantone L, et al Predictors of global methylation levels in blood DNA of healthy subjects: a combined analysis. Int J Epidemiol. 2012;41: 126–139. 10.1093/ije/dyq154 20846947 PMC3304518

[6] Zhang FF, Cardarelli R, Carroll J, Fulda KG, Kaur M, Gonzalez K, et al Significant differences in global genomic DNA methylation by gender and race/ethnicity in peripheral blood. Epigenetics. 2011;6: 623–629. 21739720 10.4161/epi.6.5.15335 PMC3230547

[7] Fraga MF, Ballestar E, Paz MF, Ropero S, Setien F, Ballestar ML, et al Epigenetic differences arise during the lifetime of monozygotic twins. Proc Natl Acad Sci U S A. 2005;102: 10604–10609. 16009939 10.1073/pnas.0500398102 PMC1174919

[8] Chowdhury S, Cleves MA, MacLeod SL, James SJ, Zhao W, Hobbs CA. Maternal DNA hypomethylation and congenital heart defects. Birth Defects Res A Clin Mol Teratol. 2011;91: 69–76. 10.1002/bdra.20761 21254366 PMC3168545

[9] Friso S, Choi SW, Girelli D, Mason JB, Dolnikowski GG, Bagley PJ, et al A common mutation in the 5,10-methylenetetrahydrofolate reductase gene affects genomic DNA methylation through an interaction with folate status. Proc Natl Acad Sci USA. 2002;99: 5606–5611. 11929966 10.1073/pnas.062066299 PMC122817

[10] Castro R, Rivera I, Ravasco P, Camilo ME, Jakobs C, Blom HJ, et al 5, 10-methylenetetrahydrofolate reductase (MTHFR) 677C–>T and 1298A–>C mutations are associated with DNA hypomethylation. J Med Genet. 2004;41: 454–458. 15173232 10.1136/jmg.2003.017244 PMC1735802

[11] Pogribny IP, Beland FA. DNA hypomethylation in the origin and pathogenesis of human diseases. Cell Mol Life Sci.2009;66: 2249–2261. 10.1007/s00018-009-0015-5 19326048 PMC11115809

[12] Robertson KD. DNA methylation and human disease. Nat Rev Genet. 2005;6: 597–610. 16136652 10.1038/nrg1655

[13] Herrera LA, Prada D, Andonegui MA, Dueñas-González A. The epigenetic origin of aneuploidy. Curr Genomics. 2008;9: 43–50. 10.2174/138920208783884883 19424483 PMC2674307

[14] Wilson AS, Power BE, Molloy PL. DNA hypomethylation and human diseases. Biochim Biophys Acta. 2007;1775: 138–162. 17045745 10.1016/j.bbcan.2006.08.007

[15] Chen RZ, Pettersson U, Beard C, Jackson-Grusby L, Jaenisch R. DNA hypomethylation leads to elevated mutation rates. Nature. 1998;395: 89–93. 9738504 10.1038/25779

[16] Ji W, Hernandez R, Zhang XY, Qu GZ, Frady A, Varela M, et al DNA demethylation and pericentromeric rearrangements of chromosome 1. Mutat Res-Fundam Mol Mech Mutagen. 1997;379: 33–41. 10.1016/s0027-5107(97)00088-2 9330620

[17] Weisenberger DJ, Campan M, Long TI, Kim M, Woods C, Fiala E, et al Analysis of repetitive element DNA methylation by MethyLight. Nucleic Acids Res. 2005;33: 6823–6836. 16326863 10.1093/nar/gki987 PMC1301596

[18] Yang AS, Estecio MR, Doshi K, Kondo Y, Tajara EH, Issa JPJ. A simple method for estimating global DNA methylation using bisulfite PCR of repetitive DNA elements. Nucleic Acids Res. 2004;32: e38 14973332 10.1093/nar/gnh032 PMC373427

[19] Woo HD, Kim J. Global DNA Hypomethylation in Peripheral Blood Leukocytes as a Biomarker for Cancer Risk: A Meta-Analysis. PLoS ONE. 2007;7: e34615. 10.1371/journal.pone.0034615 PMC3324531 22509334

[20] Kazazian HH Jr. Mobile elements: drivers of genome evolution. Science. 2004;303: 1626–1632. 15016989 10.1126/science.1089670

[21] EUROCAT [Internet]. European network of population-based registries for the epidemiologic surveillance of congenital anomalies. Available: http://www.eurocat-network.eu/ACCESSPREVALENCEDATA/PrevalenceTables. Accessed 3 June 2014.

[22] Vraneković J, Božović IB, Grubić Z, Wagner J, Pavlinić D, Dahoun S, et al Down Syndrome: Parental Origin, Recombination, and Maternal Age. Genet Test Mol Biomarkers. 2012;16: 70–73. 10.1089/gtmb.2011.0066 21861707 PMC3265771

[23] Freeman SB, Allen EG, Oxford-Wright CL, Tinker SW, Druschel C, Hobbs CA, et al The National Down Syndrome Project: design and implementation. Public Health Rep. 2007;122: 62–72. 17236610 10.1177/003335490712200109 PMC1802119

[24] James SJ, Pogribna M, Pogribny IP, Melnyk S, Hine RJ, Gibson JB, et al Abnormal folate metabolism and mutation in the methylenetetrahydrofolate reductase gene may be maternal risk factors for Down syndrome. Am J Clin Nutr. 1999;70: 495–501. 10500018 10.1093/ajcn/70.4.495

[25] Oliver TR, Feingold E, Yu K, Cheung V, Tinker S, Yadav-Shah M, et al New Insights into Human Nondisjunction of Chromosome 21 in Oocytes. PLoS Genet. 2008;4: e1000033 10.1371/journal.pgen.1000033 18369452 PMC2265487

[26] Allen EG, Freeman SB, Druschel C, Hobbs CA, O'Leary LA, Romitti PA, et al Maternal age and risk for trisomy 21 assessed by the origin of chromosome nondisjunction: a report from the Atlanta and National Down Syndrome Projects. Hum Genet. 2009;125: 41–52. 10.1007/s00439-008-0603-8 19050929 PMC2833410

[27] Buard J, de Massy B. Playing hide and seek with mammalian meiotic crossover hotspots. Trends Genet. 2007;23: 301–309. 17434233 10.1016/j.tig.2007.03.014

[28] Sigurdsson MI, Smith AV, Bjornsson HT, Jonsson JJ. HapMap methylation-associated SNPs, markers of germline DNA methylation, positively correlate with regional levels of human meiotic recombination. Genome Res. 2009;19: 581–589. 10.1101/gr.086181.108 19158364 PMC2665777

[29] Richardson BC. Role of DNA methylation in the regulation of cell function: autoimmunity, aging and cancer. J Nutr. 2002;132: 2401–2405. 12163700 10.1093/jn/132.8.2401S

[30] Colić Barić I, Satalić Z, Keser I, Cecić I, Sucić M. Validation of the folate food frequency questionnaire with serum and erythrocyte folate and plasma homocysteine. Int J Food Sci Nutr. 2009;60: S5:10–18. 19184765 10.1080/09637480802249074

[31] Coppedè F, Marini G, Bargagna S, Stuppia L, Minichilli F, Fontana I, et al Folate gene polymorphisms and the risk of Down syndrome pregnancies in young Italian women. Am J Med Genet A. 2006;140: 1083–1091. 16596679 10.1002/ajmg.a.31217

[32] Eads CA, Danenberg KD, Kawakami K, Saltz LB, Blake C, Shibata D, et al MethyLight: a high-throughput assay to measure DNA methylation. Nucleic Acids Res. 2000;15: 28(8):E32 10734209 10.1093/nar/28.8.e32 PMC102836

[33] Zeschnigk M, Böhringer S, Price EA, Onadim Z, Masshöfer L, Lohmann DR. A novel real-time PCR assay for quantitative analysis of methylated alleles (QAMA): analysis of the retinoblastoma locus. Nucl Acids Res. 2004;32(16): e125 15353561 10.1093/nar/gnh122 PMC519124

[34] Ogino S, Kawasaki T, Brahmandam M, Cantor M, Kirkner GJ, Spiegelman D, et al Precision and performance characteristics of sodium bisulfite conversion and real-time PCR (MethyLight) for quantitative DNA methylation analysis. J Mol Diagn. 2006;8: 209–217. 16645207 10.2353/jmoldx.2006.050135 PMC1867588

[35] Morris JK, Alberman E. Trends in Down's syndrome live births and antenatal diagnoses in England and Wales from 1989 to 2008: analysis of data from the National Down Syndrome Cytogenetic Register. BMJ. 2009;339: b3794 10.1136/bmj.b3794 19858532 PMC2767483

[36] Frosst P, Blom HJ, Milos R, Goyette P, Sheppard CA, Matthews RG, et al A candidate genetic risk factor for vascular disease: a common mutation in methylenetetrahydrofolate reductase. Nat Genet. 1995;10: 111–113. 7647779 10.1038/ng0595-111

[37] Guenther BD, Sheppard CA, Tran P, Rozen R, Matthews RG, Ludwig ML. The structure and properties of methylenetetrahydrofolate reductase from Escherichia coli suggest how folate ameliorates human hyperhomocysteinemia. Nat Struct Biol. 1999;6: 359–365. 10201405 10.1038/7594

[38] Lehmann EL. Nonparametrics. Statistical Methods Based on Ranks Reprinting of 1988 revision of 1975 Holden-Day ed. New York: Springer 2006. 463 p.

[39] Richards KL, Zhang B, Baggerly KA, Colella S, Lang JC, Schuller DE, et al Genome-wide hypomethylation in head and neck cancer is more pronounced in HPV-negative tumors and is associated with genomic instability. PLoS One. 2009;4: e4941 10.1371/journal.pone.0004941 19293934 PMC2654169

[40] Costello JF, Plass C. Methylation matters. J Med Genet. 2001;38: 285–303. 11333864 10.1136/jmg.38.5.285 PMC1734882

[41] Lengauer C, Kinzler KW, Vogelstein B. DNA methylation and genetic instability in colorectal cancer cells. Proc Natl Acad Sci U S A. 1997;94: 2545–2550. 9122232 10.1073/pnas.94.6.2545 PMC20125

[42] Karpf AR, Matsui S. Genetic disruption of cytosine DNA methyltransferase enzymes induces chromosomal instability in human cancer cells. Cancer Res. 2005;65: 8635–8639. 16204030 10.1158/0008-5472.CAN-05-1961

[43] Xu GL, Bestor TH, Bourc'his D, Hsieh CL, Tommerup N, Bugge M, et al Chromosome instability and immunodeficiency syndrome caused by mutations in a DNA methyltransferase gene. Nature. 1999;402: 187–191. 10647011 10.1038/46052

[44] Eden A, Gaudet F, Waghmare A, Jaenisch R. Chromosomal instability and tumors promoted by DNA hypomethylation. Science. 2003;300: 455 12702868 10.1126/science.1083557

[45] Gaudet F, Hodgson JG, Eden A, Jackson-Grusby L, Dausman J, Gray JW, et al Induction of tumors in mice by genomic hypomethylation. Science. 2003;300: 489–492. 12702876 10.1126/science.1083558

[46] Bailey LB, Gregory JF III. Folate metabolism and requirements. J Nutr. 1999;129: 779–782. 10203550 10.1093/jn/129.4.779

[47] Kluijtmans LA, Young IS, Boreham CA, Murray L, McMaster D, McNulty H, et al Genetic and nutritional factors contributing to hyperhomocysteinemia in young adults. Blood. 2003;101: 2483–2488. 12642343 10.1182/blood.V101.7.2483

[48] Coppedè F. Advances in the genetic aspects linking folate metabolism to the maternal risk of birth of a child with Down syndrome. OA Genetics. 2013;01;1(1):1.

[49] Vogt P. Potential genetic functions of tandem repeated DNA sequence blocks in the human genome are based on a highly conserved "chromatin folding code". Hum Genet. 1990;84: 301–336. 2407640 10.1007/BF00196228

[50] Lehnertz B, Ueda Y, Derijck AA, Braunschweig U, Perez-Burgos L, Kubicek S, et al Suv39h-mediated histone H3 lysine 9 methylation directs DNA methylation to major satellite repeats at pericentric heterochromatin. Curr Biol. 2003;13: 1192–1200. 12867029 10.1016/s0960-9822(03)00432-9

[51] Blasco MA. The epigenetic regulation of mammalian telomeres. Nat Rev Genet. 2007;8: 299–309. 17363977 10.1038/nrg2047

[52] Allshire RC, Karpen GH. Epigenetic regulation of centromeric chromatin: old dogs, new tricks? Nat Rev Genet. 2008;9: 923–937. 10.1038/nrg2466 19002142 PMC2586333

[53] Subirana JA, Messeguer X. The most frequent short sequences in non-coding DNA. Nucleic Acids Res. 2010;38: 1172–1181. 10.1093/nar/gkp1094 19966278 PMC2831315

[54] Bourc’his D, Bestor TH. Meiotic catastrophe and retrotransposon reactivation in male germ cells lacking Dnmt3L. Nature. 2004;431: 96–99. 15318244 10.1038/nature02886

[55] Peters AH, O'Carroll D, Scherthan H, Mechtler K, Sauer S, Schöfer C, et al Loss of the Suv39h histone methyltransferases impairs mammalian heterochromatin and genome stability. Cell. 2001;107: 323–327. 11701123 10.1016/s0092-8674(01)00542-6

[56] De La Fuente R, Baumann C, Fan T, Schmidtmann A, Dobrinski I, Muegge K. Lsh is required for meiotic chromosome synapsis and retrotransposon silencing in female germ cells. Nat Cell Biol. 2006;8: 1448–1454. 17115026 10.1038/ncb1513

[57] Pidoux AL, Allshire RC. The role of heterochromatin in centromere function. Philos Trans R Soc Lond B Biol Sci. 2005;360: 569–579. 15905142 10.1098/rstb.2004.1611 PMC1569473

[58] Lippman Z, Martienssen R. The role of RNA interference in heterochromatic silencing. Nature. 2004;431: 364–370. 15372044 10.1038/nature02875

[59] Rodriguez J, Frigola J, Vendrell E, Risques RA, Fraga MF, Morales C, et al Chromosomal instability correlates with genome-wide DNA demethylation in human primary colorectal cancers. Cancer Res. 2006;66: 8462–9468. 16951157 10.1158/0008-5472.CAN-06-0293

[60] Wilson IM, Davies JJ, Weber M, Brown CJ, Alvarez CE, MacAulay C, et al Epigenomics: mapping the methylome. Cell Cycle. 2006;5: 155–158. 16397413 10.4161/cc.5.2.2367

[61] Gonzalo S, Jaco I, Fraga MF, Chen T, Li E, Esteller M, et al DNA methyltransferases control telomere length and telomere recombination in mammalian cells. Nat Cell Biol. 2006;8: 416–424. 16565708 10.1038/ncb1386

[62] Richardson B. Impact of aging on DNA methylation. Ageing Res Rev. 2003;2: 245–261. 12726774 10.1016/s1568-1637(03)00010-2

[63] Stenvinkel P, Karimi M, Johansson S, Axelsson J, Suliman M, Lindholm B, et al Impact of inflammation on epigenetic DNA methylation—a novel risk factor for cardiovascular disease. J Intern Med. 2007;261: 488–499. 17444888 10.1111/j.1365-2796.2007.01777.x

[64] Piyathilake CJ, Badiga S, Alvarez RD, Partridge EE, Johanning GL. A lower degree of PBMC L1 methylation is associated with excess body weight and higher HOMA-IR in the presence of lower concentrations of plasma folate. PLoS One. 2013;8: e54544 10.1371/journal.pone.0054544 23358786 PMC3554730

[65] Kim M, Long TI, Arakawa K, Wang R, Yu MC, Laird PW. DNA methylation as a biomarker for cardiovascular disease risk. PLoS One. 2010;5: e9692 10.1371/journal.pone.0009692 20300621 PMC2837739

